# Does the presence of magnetic resonance imaging-detected osteitis at diagnosis with rheumatoid arthritis lower the risk for achieving disease-modifying antirheumatic drug-free sustained remission: results of a longitudinal study

**DOI:** 10.1186/s13075-018-1553-8

**Published:** 2018-04-10

**Authors:** L. E. Burgers, D. M. Boeters, M. Reijnierse, A. H. M. van der Helm-van Mil

**Affiliations:** 1 Department of Rheumatology, Leiden University Medical Center, C-01-046, PO Box 9600, 2300 RC Leiden, The Netherlands; 2 Department of Radiology, Leiden University Medical Center, Leiden, the Netherlands; 3 Department of Rheumatology, Erasmus Medical Center, Rotterdam, the Netherlands

**Keywords:** Magnetic resonance imaging, Rheumatoid arthritis, Early rheumatoid arthritis, Outcomes research

## Abstract

**Background:**

Although infrequent, some rheumatoid arthritis (RA) patients achieve disease-modifying antirheumatic drug (DMARD)-free sustained remission. The absence of RA-specific autoantibodies, such as anticitrullinated protein antibodies (ACPA), is known to be associated with this outcome but further mechanisms underlying the chronic nature of RA are largely unknown. Magnetic resonance imaging (MRI)-detected bone marrow edema (BME), or osteitis, strongly predicts erosive progression and is associated with ACPA positivity. Therefore, we hypothesized that the presence of MRI-detected osteitis is also predictive of not achieving DMARD-free sustained remission and that the presence of osteitis mediates the association between ACPA and DMARD-free sustained remission.

**Methods:**

A 1.5 T unilateral hand and foot MRI was performed at disease presentation in 238 RA patients, evaluating BME, synovitis, and tenosynovitis (summed as MRI inflammation score). DMARD-free sustained remission, defined as the absence of clinical synovitis after DMARD cessation that persisted during the total follow-up, was assessed (median follow-up 3.8 years). Associations between the different MRI-detected inflammatory features and this outcome were studied. A mediation analysis was performed to study whether the presence of BME mediated the association between ACPA and DMARD-free sustained remission. Finally, patterns of MRI-detected inflammation with regard to DMARD-free sustained remission were studied using partial least squares (PLS) regression.

**Results:**

Forty-six (19.3%) patients achieved DMARD-free sustained remission. ACPA positivity associated independently with remission (hazard ratio (HR) 0.16, 95% confidence interval (CI) 0.06–0.39). In contrast, no associations were observed between MRI-detected BME (HR 0.99, 95% CI 0.94–1.03), or other MRI inflammatory features, and achieving DMARD-free sustained remission. Thus, the presence of BME did not mediate the association between ACPA and DMARD-free sustained remission. Furthermore, a PLS analysis revealed that patients who did or did not achieve remission could not be distinguished by patterns of MRI-detected inflammation.

**Conclusions:**

At disease presentation, osteitis, as well as other MRI-detected inflammatory features, was not associated with achieving DMARD-free sustained remission over time. Thus, imaging predictors for joint damage and disease persistence differ. The processes mediating RA chronicity remain largely unsolved.

**Electronic supplementary material:**

The online version of this article (10.1186/s13075-018-1553-8) contains supplementary material, which is available to authorized users.

## Background

As joint damage is becoming less prevalent in patients with rheumatoid arthritis (RA), the focus is shifting to other disease outcomes such as disease-modifying antirheumatic drug (DMARD)-free sustained remission [1]. DMARD-free sustained remission means that DMARDs can be discontinued without the recurrence of clinical synovitis [1, 2]. Thanks to improved treatment strategies, DMARD-free sustained remission is more feasible and corresponds with perceived normalization of physical functioning and pain, fatigue, and stiffness levels for patients [1, 3].

Pathophysiological mechanisms behind RA chronicity or, the opposite, DMARD-free sustained remission are largely unknown [4]. Short symptom duration at disease presentation has been shown to be predictive suggesting that the timing of disease modification is important [1, 2, 5]. The strongest predictor for DMARD-free sustained remission is the absence of RA-specific autoantibodies, such as anticitrullinated protein antibodies (ACPA) [1, 2]. This suggests that patients who can achieve this outcome are inherently different.

Joint destruction and not achieving DMARD-free sustained remission are both characteristics of a severe disease course. Therefore, hypothetically, predictors for both outcomes are similar. ACPA are such an example. However, local joint inflammation, measured using the swollen joint count (SJC), while predictive for erosive progression [6, 7], was not predictive for DMARD-free sustained remission [1, 2]. A more sensitive method to detect local inflammation is magnetic resonance imaging (MRI). In addition to synovitis and tenosynovitis, MRI can detect bone marrow edema (BME), also called osteitis. Osteitis is not specific for RA as it has also been observed in other conditions such as psoriatic arthritis and in symptom-free controls [8–10]. In RA, the presence of osteitis is strongly predictive for erosive progression, with odds ratios reported up to 60 [8, 11–13]. The process of joint destruction is likely to be ACPA-driven since ACPA-positive RA patients have higher osteitis scores [14, 15]. Furthermore, ACPA have been shown to be able to induce osteoclastogenesis leading to subsequent bone loss [16] and a histologic study of bone samples in which MRI-detected osteitis is present revealed an increased presence of osteoclasts compared with control samples [17]. Thus, osteitis appears to be a mediator in the link between ACPA and erosive progression.

Our ultimate aim is to increase the understanding of processes involved in achieving DMARD-free sustained remission or disease chronicity. With the underlying assumption that predictors for erosive progression and not achieving DMARD-free sustained remission are similar, we hypothesized that the presence of osteitis at disease presentation also associates with a decreased chance of achieving DMARD-free sustained remission. Furthermore, we hypothesized that the association between ACPA and DMARD-free sustained remission is mediated by osteitis. To evaluate these hypotheses, associations between MRI-detected inflammation and DMARD-free sustained remission were evaluated and a mediation analysis was performed. Finally, to study not only the presence of osteitis itself, but also its relationship with other types of MRI-detected inflammation, and its location, and achieving DMARD-free sustained remission, we looked at whether a pattern of MRI-detected inflammation (covering the type of MRI inflammatory feature, severity, and location) could distinguish patients who did and did not achieve this outcome over time.

## Methods

### Patients

**Fig. 1:**
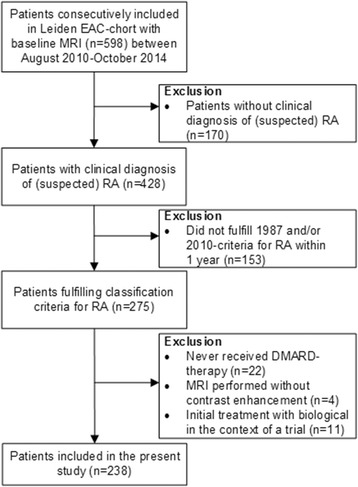
Flowchart of patient selection. DMARD disease modifying antirheumatic drug, EAC early arthritis clinic, MRI magnetic resonance imaging, RA rheumatoid arthritis

The Leiden Early Arthritis Clinic (EAC) is a population-based inception cohort that includes patients with clinically confirmed synovitis of ≥ 1 joint and a symptom duration of < 2 years [18]. Between August 2010 and September 2014 an MRI was performed at baseline in 598 consecutive patients. Of these patients, 275 fulfilled the 1987 and/or 2010 criteria for RA and were treated with DMARDs during the first year. Thirty-seven patients were excluded (22 because they never received DMARD therapy despite fulfilling classification criteria, 4 because the MRI was performed without contrast, and 11 because of participation in a trial with initial biologic therapy that differed from routine care) [19, 20]. Thus, in total 238 RA patients were studied (Fig. 1). According to local and national guidelines, RA patients were initially treated with methotrexate (started at or after the 2-week visit); after failure, another conventional DMARD was initiated or added. Biologicals were allowed after failure of > 2 conventional DMARDs and were used in 22 patients  (9%) of whom none achieved DMARD-free sustained remission. Treatment was steered by disease activity score (DAS). All patients had scheduled EAC visits at 4 months, 8 months, 12 months, 18 months, 24 months, and then yearly. Besides these visits, regular visits were planned when deemed necessary by either the patient or rheumatologist (usually every 3–4 months). All patients provided written informed consent. The study was approved by the local medical ethical committee.

### MRI

A unilateral 1.5 T MRI of the metacarpophalangeal (MCP; 2–5) joints, wrist, and metatarsophalangeal (MTP; 1–5) joints of the most painful side or, in case of equally severe symptoms, of the dominant side was made < 2 weeks after inclusion. MRIs were scored on BME and synovitis in line with the RA MRI scoring (RAMRIS) method [21] and on tenosynovitis according to Haavardsholm et al. [22]. The total MRI inflammation score was the sum of total BME, synovitis, and tenosynovitis scores. MRIs were scored by two experienced readers (intraclass correlation coefficient (ICC) 0.94) who were blinded to clinical data. The mean scores of the two readers were taken. More information about the MRI scanning protocol and scoring method has been described previously [10, 23, 24] and is provided in Additional file 1 (Supplementary methods).

### DMARD-free sustained remission

Medical records of all patients were reviewed in April 2017 for the presence of DMARD-free sustained remission, defined as the absence of clinical synovitis that persisted after cessation of DMARDs during the total subsequent follow-up. The follow-up duration after DMARD cessation had to be ≥ 1 year to fulfill the definition. The date of remission was the date 1 year after DMARD cessation. For patients not achieving remission, the date of censoring was the date of reviewing the medical records or an earlier date in case patients were lost to follow-up.

### Statistical analysis

Associations between baseline characteristics, including MRI-detected inflammatory features and DMARD-free sustained remission, were assessed using univariable and multivariable Cox regression analyses. Partial least squares (PLS) regression was used to study whether certain patterns of MRI-detected inflammation discriminated between patients who did and did not achieve remission. This was done since not only the single presence of BME but also information on its presence in combination with other MRI-detected inflammatory features, or the location of BME, could be discriminative. Previous studies observed that BME is often present in certain carpal bones of symptom-free controls (such as the lunate) which may be caused by mechanical factors, and suggests that the location of BME can be relevant for the interpretation [10]. PLS regression clusters features and locations of MRI-detected inflammation that frequently occur together into factors, and presents for each factor the variance in the outcome that is explained by this factor. These factors can then be plotted against each other to look for clustering. A mediation analysis was performed according to Baron and Kenny [25]. Detailed statistical information is provided in Additional file 1. SPSS V.23.0 software was used.

## Results

### Patient characteristics

Of the 238 studied patients, 46 (19.3%) achieved DMARD-free sustained remission after a median duration of 2.8 (interquartile range (IQR) 2.0–3.5) years. For patients who did not achieve DMARD-free sustained remission, the median follow-up duration was 3.8 (IQR 3.0–5.0) years. Table 1 presents the baseline characteristics. The majority of patients were female and the mean age was 57 years. Associations between clinical characteristics and achieving DMARD-free sustained remission are shown in Table 2. Only the presence of ACPA was independently associated (hazard ratio (HR) 0.16, 95% confidence interval (CI) 0.06–0.39) with DMARD-free sustained remission.

**Table 1:** Baseline clinical and MRI characteristics of all patients and separately for patients that have and have not achieved DMARD-free sustained remission

	All patients (*n* = 238)	DMARD-free sustained remission (*n* = 46)	No remission (*n* = 192)
Clinical characteristics			
Age in years, mean (SD)	57 (14)	62 (13)	55 (14)
Female gender, *n* (%)	157 (66)	28 (61)	129 (67)
Symptom duration in weeks	15 (7–32)	14 (5–21)	17 (7–34)
Symptom duration > 12 weeks, *n* (%)	100 (42)	20 (44)	80 (42)
HAQ score, mean (SD)	0.9 (0.6)	1.1 (0.5)	1.0 (0.9)
68-TJC	8 (4–14)	9 (5–17)	8 (4–13)
66-SJC	5 (2–10)	8 (3–13)	5 (2–10)
CRP mg/L	9 (3–21)	17 (9–27)	8 (3–20)
ESR mm/h, mean (SD)	30 (24)	33 (25)	29 (24)
ACPA-positive, *n* (%)	120 (50)	6 (13)	114 (59)
RF-positive, *n* (%)	140 (59)	16 (35)	124 (65)
MRI characteristics			
BME score	3.5 (1.0–8.0)	4.0 (1.4–7.3)	3.5 (1.0–8.5)
Synovitis score	5.0 (2.5–9.5)	7.3 (3.0–10.0)	4.5 (2.5–9.5)
Tenosynovitis score	4.0 (1.5–7.5)	4.5 (1.8–9.3)	4.0 (1.5–7.0)
Total MRI inflammation score	13.5 (6.5–26.0)	16.5 (6.4–27.6)	12.5 (6.5–25.5)

All values are shown as median (interquartile range) unless indicated otherwise

Values are missing as follows (*n*): symptom duration (2), HAQ (13), TJC (11), SJC (12), and ESR (2)

*ACPA* anticitrullinated protein antibodies, *BME* bone marrow edema, *CRP* C-reactive protein, *DMARD* disease-modifying antirheumatic drug, *ESR* erythrocyte sedimentation rate, *HAQ* health assessment questionnaire, *MRI* magnetic resonance imaging, *RF* rheumatoid factor, *SD* standard deviation; *SJC* swollen joint count, *TJC* tender joint count

**Table 2:** Results of univariable and multivariable Cox regression analyses of clinical baseline characteristics in relation to achievement of DMARD-free sustained remission

	Univariable HR (95% CI)	Multivariable HR (95% CI)^a^
Clinical characteristics		
Age in years	1.04 (1.02–1.07)*	1.02 (0.99–1.04)
Female gender	0.81 (0.45–1.47)	
Symptom duration > 12 weeks	0.83 (0.46–1.50)	
HAQ score	1.11 (0.69–1.78)	
68-TJC	1.02 (0.98–1.05)	
66-SJC	1.06 (1.02–1.10)*	1.03 (0.99–1.08)
CRP mg/L	1.01 (1.0–1.02)*	1.00 (0.99–1.01)
ESR mm/h	1.01 (1.0–1.02)	
ACPA-positive	0.12 (0.05–0.27)*	0.16 (0.06–0.39)*
RF-positive	0.33 (0.18–0.61)*	0.80 (0.41–1.6)

*ACPA* anticitrullinated protein antibodies, *CI* confidence interval, *CRP* C-reactive protein, *DMARD* disease-modifying antirheumatic drug, *ESR* erythrocyte sedimentation rate, *HAQ* health assessment questionnaire, *HR* hazard ratio, *RF* rheumatoid factor, *SJC* swollen joint count, *TJC* tender joint count

*p < 0.05

^a^The multivariable model includes all variables that showed a significant association with sustained DMARD-free remission in univariable Cox regression analyses. A total of 224 patients were included in the multivariable model since data on the tested variables were incomplete for 14 patients. No statistically significant differences were present between patients with missing data and the patient included in the multivariable model (data not shown)

### Association between MRI-detected inflammatory features and DMARD-free sustained remission

**Fig. 2:**
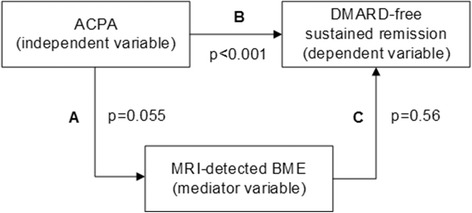
Schematic overview of the causal path between ACPA and DMARD-free sustained remission that was studied using a mediation model as described by Baron and Kenny [25]. According to the Baron and Kenny model there are three requirements for a true mediation relationship. The first step is to regress the dependent variable on the independent variable to confirm that the independent variable is predictive for the dependent variable. In our data, the absence of anticitrullinated protein antibodies (ACPA) was indeed predictive for disease-modifying antirheumatic drug (DMARD)-free sustained remission (HR 0.16, *p* < 0.001). The second step is to regress the mediator variable on the independent variable to confirm that the independent variable is a predictor of the mediator. The association between ACPA and bone marrow edema (BME) was not statistically significant although there was a strong tendency towards significance (β = 1.58, *p* = 0.055). The final step would be to regress the dependent variable on both the mediator and independent variable to confirm that the mediator is a significant predictor of the dependent variable and the effect of the independent variable from the first step is greatly reduced. This was not the case (HR for ACPA corrected for BME 0.12, *p* < 0.001). As shown in Table 2, BME scores were not associated with DMARD-free sustained remission (HR 0.99, *p* = 0.56). Thus, this mediation analysis revealed that magnetic resonance imaging (MRI)-detected BME does not mediate the association of ACPA with not achieving DMARD-free sustained remission

The MRI-detected BME score was not associated with achieving DMARD-free sustained remission (HR 0.99, 95% CI 0.94–1.03). This was the same for MRI-detected synovitis (HR 1.03, 95% CI 0.97–1.09), tenosynovitis (HR 1.04, 95% CI 0.99–1.10), and the total MRI inflammation score (HR 1.01, 95% CI 0.99–1.03) (Table 3). Also, in multivariable models where corrections were made for the simultaneous presence of other inflammatory features and ACPA, BME was not associated with remission (Table 3). Mediation analysis revealed that BME did not mediate the association between ACPA and DMARD-free sustained remission (Fig. 2).

**Table 3:** Associations between MRI-detected inflammation and achieving DMARD-free sustained remission over time

Type of MRI-detected inflammation	Univariable model HR (95% CI)	Multivariable model 1^a^ HR (95% CI)	Multivariable model 2^b^ HR (95% CI)
BME	0.99 (0.94–1.03)	0.96 (0.99–1.02)	1.00 (0.94–1.07)
Synovitis	1.03 (0.97–1.09)	1.04 (0.95–1.15)	1.00 (0.90–1.10)
Tenosynovitis	1.04 (0.99–1.10)	1.03 (0.95–1.11)	1.01 (0.94–1.08)
Total inflammation	1.01 (0.99–1.03)	–	–

All HRs are per point increase of MRI-detected inflammation

*BME* bone marrow edema, *CI* confidence interval, *DMARD* disease-modifying antirheumatic drug, *HR* hazard ratio, *MRI* magnetic resonance imaging

^a^Multivariable model 1 includes MRI-detected BME, synovitis and tenosynovitis

^b^Multivariable model 2 includes MRI-detected BME, synovitis and tenosynovitis as well as ACPA

### Patterns of MRI-detected inflammation

**Fig. 3:**
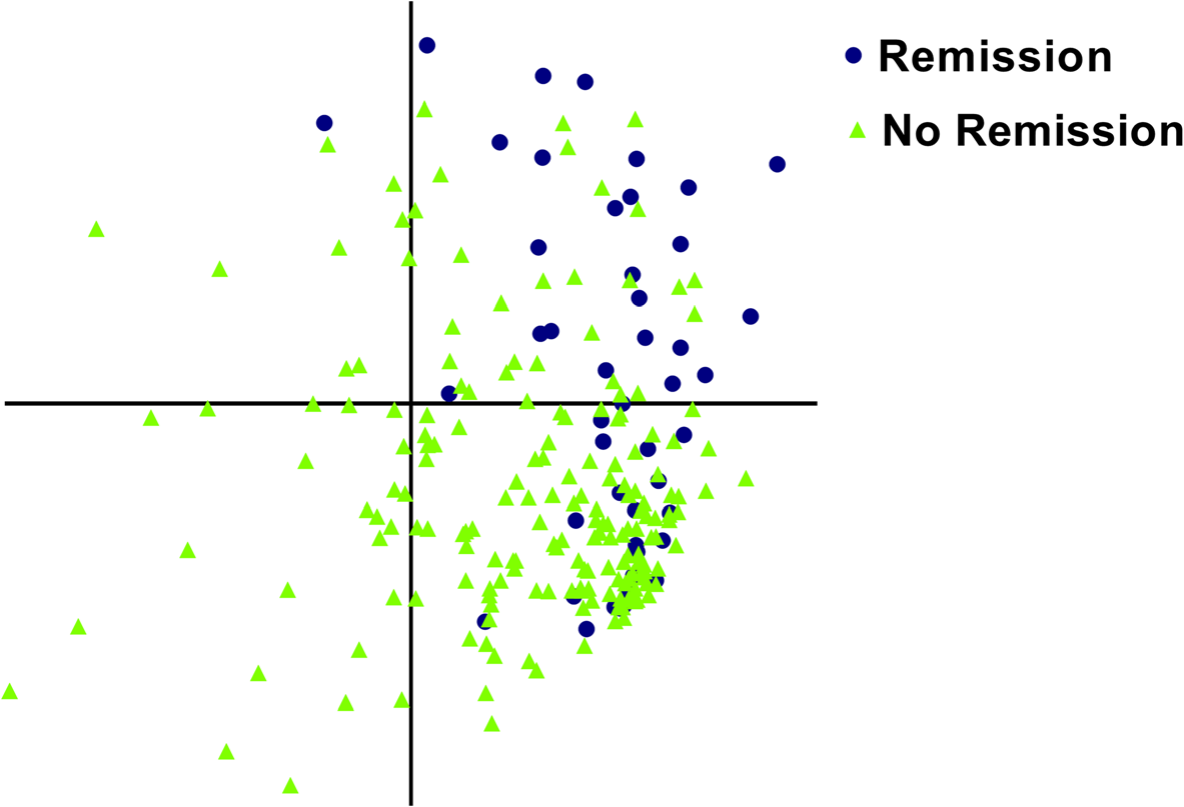
A combination of MRI features (covering type, location, and severity of inflammation) did not cluster RA patients who did or did not achieve DMARD-free sustained remission over time. This figure depicts the results of a PLS regression analysis. Two factors were identified that together explained 15.0% of the explained variance between patients who did and did not achieve DMARD-free sustained remission over time. Individual patient scores on factor 1 (*y* axis) and factor 2 (*x* axis) are plotted against each other. The figure reveals that there is overlap, which means that there are no clear patterns of MRI-detected inflammation that discriminated between patients who did and did not achieve DMARD-free sustained remission

Finally, we questioned whether a combination of features (covering the inflammatory features, the severity, and the location of MRI-detected inflammation), not a single inflammatory feature, was able to distinguish between patients who did and did not achieve DMARD-free sustained remission. Using PLS, two factors were identified that together explained 15.0% of the variance. When these factors were plotted against each other, no clear clusters were observed. Thus, no patterns of MRI-detected inflammation were identified that were associated with achieving DMARD-free remission (Fig. 3).

## Discussion

Mechanisms underlying (the resolution of) the chronic nature of RA are scarcely understood. Moreover, there are few known predictors for DMARD-free sustained remission. Although MRI-detected inflammation, and osteitis in particular, is a potent predictor for erosive progression [8, 11, 13], this imaging marker was not predictive for DMARD-free sustained remission.

The absence of this association was surprising since ACPA has been associated with higher osteitis scores [14, 15] and less DMARD-free sustained remission [2]. Although our data showed an association between ACPA and osteitis, there was no association between osteitis and achieving DMARD-free sustained remission. Therefore, osteitis did not mediate the association of ACPA with DMARD-free sustained remission (Fig. 2).

Thus, the studied imaging predictors for erosive progression and RA chronicity differed. Previous studies on genetic factors also showed that, although *IL2RA* and *HLA-DRB1*-encoding shared epitope alleles were risk factors for both outcomes, the majority of genetic factors associated with erosive progression were not associated with RA persistence [26, 27]. Together, this suggests that pathophysiological mechanisms underlying erosive progression and RA persistence are different. Thus, our main hypothesis, namely that imaging predictors for both outcomes are similar, is likely unjust. So far, only the absence of autoantibodies and short symptom duration have been shown to be predictive for DMARD-free sustained remission. Besides these factors, there are probably other, yet unknown, host and environmental factors that play pivotal roles in processes underlying disease resolution.

This study evaluated MRI-detected inflammation at diagnosis in relation to achieving DMARD-free sustained remission over time. Thus, the hypothesis that was tested presumed that patients that achieved remission were already different in respect to joint inflammation in an early, DMARD-naive disease phase. Our study did not contain longitudinal MRI data. Although we previously observed that osteitis is often a persisting feature [6], we did not perform an MRI at the time of remission. Others observed, in DMARD-treated patients in DAS remission, that residual inflammation was present in the majority of patients (up to 96% for synovitis) [28–30]. The clinical relevance of residual inflammation is unclear, although it is suggested that its presence in patients that still receive DMARD treatment is associated with ongoing erosive progression [30]. While our definition of remission was stricter than those used in the cited studies, we do not know whether MRI-detected synovitis is present in patients that achieved DMARD-free sustained remission. Low-grade synovitis has also been observed in symptom-free patients [10] and, as our defined status of sustained DMARD-free remission corresponds with normalized levels of function, fatigue, and pain [1, 3], it is questionable if remaining MRI-detected synovitis, if present after having achieved DMARD-free sustained remission, is clinically relevant.

Patients included in this study fulfilled either the 1987 and/or 2010 criteria for RA. If not used correctly, the 2010 criteria could lead to misclassification of patients with RA, thereby influencing the study results [31]. To prevent misclassification, the classification criteria were used as intended, namely only in patients with a clinical (suspected) diagnosis of RA. Furthermore, patients who never received DMARD therapy were excluded (Fig. 1). When analyses were repeated in patients fulfilling the 1987 criteria for RA (*n* = 186), results remained similar (data not shown).

However, this association was not significant in multivariable analysis was surprising as this was not observed in two previous studies [1, 2]. However, this association was significant in multivariable analysis. Since local inflammation measured by sensitive imaging was also not associated with DMARD-free remission, the overall conclusion is that the extent of local inflammation at first presentation is not related to the likelihood of achieving DMARD-free remission.

One limitation of our study is that, despite applying a stringent definition of remission where clinical synovitis had to be absent ≥ 1 year after DMARD cessation, the available follow-up might be insufficient to detect flares after several years of follow-up. Another limitation is the sample size; although large for an MRI study, it is unknown if results would have been different in an even larger dataset. However**,** there was not even a tendency towards an association between BME and remission.

A strength of this study is that the data were obtained in a center in which DAS-steered treatment regimens imply that in case of DAS < 2.4 DMARDs are tapered and subsequently stopped. This strategy contributed to the relative high percentage of patients (19%) that achieved DMARD-free sustained remission. Other studies with varying follow-up durations reported ranges of DMARD-free remission between 3.6% and 22% [4]. Nonetheless, despite local protocols, rheumatologists had the freedom not to taper medication in patients in DAS remission. Therefore, it is possible that the current percentage is an underestimation.

## Conclusions

In conclusion, the presence of MRI-detected osteitis at the time of presentation with RA, although shown to be strongly predictive for erosive progression, was not associated with lower hazards on achieving DMARD-free sustained remission. Additionally, the current data demonstrate that MRI is not helpful in identifying patients who can achieve DMARD-free sustained remission over time. Finally, although acknowledging that the processes involved in joint damage are different from those mediating RA chronicity, the pathophysiology of the latter remains elusive.

## Additional file

Additional file 1: Supplementary methods. (DOCX 25 kb)

## Abbreviations

ACPA: Anticitrullinated protein antibodies
BME: Bone marrow edema
CI: Confidence interval
DAS: Disease activity score
DMARD: Disease-modifying antirheumatic drug
EAC: Early Arthritis Clinic
HR: Hazard ratio
IQR: Interquartile range
MCP: Metacarpophalangeal
MRI: Magnetic resonance imaging
MTP: Metatarsophalangeal
PLS: Partial least squares
RA: Rheumatoid arthritis
RAMRIS method: RA MRI scoring
SJC: Swollen joint count
