# Supplementary material for: Does the presence of magnetic resonance imaging-detected osteitis at diagnosis with rheumatoid arthritis lower the risk for achieving disease-modifying antirheumatic drug-free sustained remission: results of a longitudinal study
Source: Arthritis Res Ther. 2018 Apr 10;20:68. doi: 10.1186/s13075-018-1553-8 (PMC5894211; doi:10.1186/s13075-018-1553-8)
Supplement: Supplementary file 1 — Supplementary methods. (DOCX 25 kb) [file 13075_2018_1553_MOESM1_ESM.docx]

# Additional file 1

**Supplementary methods**

**MRI**

MR imaging was performed on a MSK-extreme 1.5T extremity MR imaging system (GE, Wisconsin, USA) using a 145mm coil for the foot and a 100mm coil for the hand. The patient was positioned in a chair beside the scanner, with the hand or foot fixed in the coil with cushions.

In the hand (MCP2-5 and wrist) the following sequence was acquired before contrast administration: T1-weighted fast spin-echo (FSE) sequence in the coronal plane (repetition time (TR) 575 ms, echo time (TE) 11.2 ms, acquisition matrix 388×288, echo train length (ETL) 2). After intravenous injection of gadolinium contrast (gadoteric acid, Guerbet, Paris, France, standard dose of 0.1 mmol/kg) the following sequences were obtained: T1-weighted FSE sequence with frequency selective fat saturation (fatsat) in the coronal plane (TR/TE 700/9.7ms, acquisition matrix 364×224, ETL 2), T1-weighted FSE fatsat sequence in the axial plane (wrist: TR/TE 540/7.7 ms; acquisition matrix 320x192; ETL 2 and MCP-joints: TR/TE 570/7.7 ms; acquisition matrix 320x192; ETL 2).

The obtained sequences of the forefoot (MTP1-5 joints) were for the first 135 patients before contrast administration: T1-weighted FSE sequence in the axial plane (TR/TE 650/17ms; acquisition matrix 388x288, ETL 2); and T2-weighted FSE fatsat sequence in the axial plane (TR/TE 3000/61.8; acquisition matrix 300x224, ETL 7). Imaging of the foot was initially limited to pre-contrast axial sequences. For the later 103 patients post-contrast sequences were included: T1-weighted FSE fatsat sequence in the axial plane (TR/TE 700/9.5ms; acquisition matrix 364x224, ETL 2) and: T1-weighted FSE fatsat sequence in the coronal plane (perpendicular to the axis of the metatarsals) (TR/TE 540/7.5ms; acquisition matrix 320x192, ETL 2).

In the MTP-joints a total MRI-inflammation score of 45 was possible (synovitis in MTP1-5 with a maximum of 3 per location and BME proximal and distal in MTP1-5 with a maximum of 3 per location). In the MCP-joints a total MRI-inflammation score of 60 was possible (synovitis in MCP2-5 with a maximum of 3 per location, BME proximal and distal in MCP2-5 with a maximum of 3 per location and tenosynovitis on the flexor and extensor side of MCP2-5 with a maximum of 3 per location). In the wrist-joint a maximum MRI-inflammation score of 84 was possible (synovitis in the radio-ulnar-, radio-carpal- and intercarpal-CMC joint with a maximum of 3 per location, BME in all the carpals and the bases of all the metacarpals and the distal radius and ulna with a maximum of 3 per location and tenosynovitis in the 6 extensor compartments and 4 flexor compartments with a maximum of 3 per location[1]). The total MRI-inflammation score therefore ranges between 0 and 189.

We used the contrast enhanced T1-weighted fat suppressed sequence to assess BME in the MCP-joints of all 238 patients. In the MTP-joints BME was assessed on T2-weighted fatsat sequences in the first 135 patients and on the contrast enhanced T1-weighted fat suppressed sequence in the latter 103 patients. According to the RAMRIS-method, T2-weighted fat suppressed sequences, or when this sequence is not available a short tau inversion recovery (STIR) sequence, should be used to assess BME. However, three previous studies have demonstrated that a contrast enhanced T1-weigthed fat suppressed sequence has a strong correlation with T2-weighted fat suppressed sequences[2–4]. Furthermore, the arthritis subcommittee of the European Society of Musculoskeletal Radiology (ESSR) also recommends the use of contrast enhanced T1-weighted fat suppressed sequences for depicting BME[5] The T2-weighted image shows increased water signal and a contrast-enhanced T1-weighted sequence shows increased water content and the increased perfusion and interstitial leakage. A strong correlation has been shown in arthritis patients and in patients without inflammatory diseases such as bone bruises, intraosseous ganglions, bone infarcts and even nonspecific cases[3,4]. Based on these results BME was assessed on contrast enhanced T1-weighted fat suppressed sequences as it has a higher signal to noise ratio and allowed a shorter scan time for patients. In addition, because T2-weighted fat suppressed sequences could be omitted, coronal sequences of the foot could be added. In total this resulted in a shorter total scan time and more information.

Field-of-view was 100mm for the hand and 140mm for the foot. Coronal sequences of the hand had 18 slices with a slice thickness of 2mm and a slice gap of 0.2mm. Coronal sequences of the foot had 20 slices with a slice thickness of 3mm and a slice gap of 0.3mm. All axial sequences had a slice thickness of 3mm and a slice gap of 0.3mm with 20 slices for the wrist, 16 for the metacarpophalangeal-joints and 14 for the foot.

**Statistics**

First the association between baseline characteristics and DMARD-free sustained remission was studied with univariable Cox proportional-hazard regression analyses. All baseline characteristics that showed a significant association were then entered in a multivariable Cox regression analysis. As there were 46 patients who achieved DMARD-free sustained remission, no more than 5 variables were entered in a multivariable model to prevent overfitting of the model. This coincided with the observation that in current analyses only 5 variables were significantly associated in univariable analysis.

Next, associations between different types of MRI-detected inflammation as well as the total inflammation score and DMARD-free sustained remission were studied using univariable Cox regression analyses. As different types of MRI-detected inflammation can frequently occur together, the different types of MRI-detected inflammation (BME, synovitis and tenosynovitis) were entered together in a multivariable model. In the next multivariable model, also clinical characteristics that were independently associated with DMARD-free sustained remission (ACPA) were entered together with the different types of MRI-detected inflammation.

A mediation analysis was performed to study whether the association between ACPA and DMARD-free sustained remission was mediated by the presence of BME. This was performed according to the model by Baron and Kenny[6].

To explore whether certain patterns of MRI-detected inflammation discriminated between patients with and without DMARD-free sustained remission, partial least square (PLS) regression analysis was used. PLS is a method of data shrinkage. PLS combines variables that often occur together in patients with a certain outcome (in this case DMARD-free sustained remission) into latent factors. The identified latent factors explain part of the variance between patients with and without this outcome. Individual patient scores on these factors can be plotted against each other to look for clustering. If clusters are observed, variables that are of importance in the latent factors (variables with a variable importance in projection>1) can be identified. Thus, a pattern of variables (in this case types and locations of MRI-detected inflammation), can be identified that for example frequently occurs in patients with DMARD-free sustained remission. However, in this analysis no clusters were observed (Figure 1).

**REFERENCES**

1. Haavardsholm EA, Østergaard M, Ejbjerg BJ, Kvan NP, Kvien TK. Introduction of a novel magnetic resonance imaging tenosynovitis score for rheumatoid arthritis: reliability in a multireader longitudinal study. Ann. Rheum. Dis. 2007;66:1216–20.

2. Stomp W, Krabben A, van der Heijde D, Huizinga TWJ, Bloem JL, van der Helm-van Mil AHM, et al. Aiming for a shorter rheumatoid arthritis MRI protocol: can contrast-enhanced MRI replace T2 for the detection of bone marrow oedema? Eur. Radiol. 2014;24:2614–22.

3. Schmid MR, Hodler J, Vienne P, Binkert CA, Zanetti M. Bone Marrow Abnormalities of Foot and Ankle: STIR versus T1-weighted Contrast-enhanced Fat-suppressed Spin-Echo MR Imaging. Radiology. 2002;224:463–9.

4. Mayerhoefer ME, Breitenseher MJ, Kramer J, Aigner N, Norden C, Hofmann S. STIR vs. T1-weighted fat-suppressed gadolinium-enhanced MRI of bone marrow edema of the knee: Computer-assisted quantitative comparison and influence of injected contrast media volume and acquisition parameters. J. Magn. Reson. Imaging. 2005;22:788–93.

5. Sudoł-Szopińska I, Jurik A, Eshed I, Lennart J, Grainger A, Østergaard M, et al. Recommendations of the ESSR Arthritis Subcommittee for the Use of Magnetic Resonance Imaging in Musculoskeletal Rheumatic Diseases. Semin. Musculoskelet. Radiol. 2015;19:396–411.

6. Baron RM, Kenny DA. The moderator-mediator variable distinction in social psychological research: conceptual, strategic, and statistical considerations. J. Pers. Soc. Psychol. 1986;51:1173–82.
